# Prevalence and sociodemographic correlates of overweight and obesity among university students in Islamabad and Rawalpindi, Pakistan: a cross-sectional study

**DOI:** 10.3389/fpubh.2026.1895592

**Published:** 2026-09-17

**Authors:** Abdul Momin Rizwan Ahmad, Zoha Imtiaz Malik, Saira Zafar, Syed Hassan Bin Umsan Shah, Rida Fatima Saeed, Umar Farooq, Juweria Abid, Sehar Iqbal, Muhammad Irfan, Saima Iqbal, Saleh A. Alsanie

**Affiliations:** 1 Department of Human Nutrition and Dietetics, NUST School of Health Sciences, National University of Sciences and Technology, Islamabad, Pakistan; 2 Department of Health Sciences, University of York, York, United Kingdom; 3 School of Public Health, Health Services Academy, Islamabad, Pakistan; 4 Department of Public Health, PRIME Consulting, Islamabad, Pakistan; 5 Department of Biotechnology, National University of Medical Sciences, Rawalpindi, Pakistan; 6 Department of Nutrition and Dietetics, National University of Medical Sciences, Rawalpindi, Pakistan; 7 College of Pharmacy, Al Ain University, Abu Dhabi, United Arab Emirates; 8 Department of Statistics, Liaquat National Hospital and Medical College, Karachi, Sindh, Pakistan; 9 Department of Basic Health Sciences, College of Applied Medical Sciences, Qassim University, Buraydah, Saudi Arabia; 10 Department of Clinical Nutrition, Medical City, Qassim University, Buraydah, Saudi Arabia

**Keywords:** cross-sectional study, obesity, overweight, Pakistan, prevalence, public health nutrition, socio-demographic correlates, university students

## Abstract

**Introduction:**

Overweight and obesity have emerged as a public health concern, particularly among university students. This increasing prevalence is linked with unhealthy lifestyles consisting of poor dietary choices, and physical inactivity, most commonly reported among students.

**Objective:**

The current study aimed to determine the prevalence of both overweight and obesity, and their socio-demographic correlates within the twin cities (Islamabad and Rawalpindi), Pakistan.

**Methods:**

A cross-sectional study was conducted among 502 students, selected via convenience sampling, enrolled in public and private universities in the twin cities. Data was collected on socio-demographic correlates and lifestyle factors, using a structured questionnaire. Data was analyzed using Statistical Package for the Social Sciences (SPSS) version 27, and chi-square test, binary logistic regression, and adjusted odds ratio, were used to determine associations between sociodemographic variables, lifestyle factors and obesity.

**Results:**

The mean age was 21.87 ± 2.27 years, while 80.7% were females. Based on Asian cut-offs for Body Mass Index (BMI), 25.5% were underweight (<18.5 kg/m^2^), 15.3% were overweight (23.0 to 27.4 kg/m^2^), and 12.9% were obese (27.5 kg/m^2^ and above). Gender (*p* < 0.001), monthly household income (*p* = 0.043), maternal education (*p* = 0.030) and having a medical condition (*p* = 0.025) were all significantly associated with obesity. However, once adjusted, only gender (adjusted odds ratio (aOR = 0.428), CI: 0.236–0.776, *p* = 0.005) remained significantly associated with obesity, with female sex depicting lower odds of obesity compared with male sex.

**Conclusion:**

Our study found a significant proportion of the study participants to be overweight (15.3%) and obese (12.9%). Gender, household income, maternal education, and having a prior medical condition, all emerged as significant associated factors of obesity in the current study. Primary prevention interventions aimed at university settings are vital to prevent and manage obesity risk factors and hence combat the increasing overweight and obesity trends among university students.

## 1 Introduction

The prevalence of obesity is increasing rapidly around the whole world. Recent studies globally have reported that the numbers of overweight females has doubled while the number of overweight males has increased fourfold. Hence making obesity a major public health concern in both developed and developing countries (1). Obesity is characterized by an abnormal buildup of fat in adipose tissue which leads to adverse health effects. Body mass index (BMI) is the most commonly used indicator to classify overweight and obesity in adults worldwide. Individuals with a BMI between 25 and 29.9 kg/m2 are considered overweight, while individuals with a BMI ≥ 30 kg/m2 are considered obese. In addition, obesity is further divided into three severity levels: class I (BMI 30.0–34.9), class II (BMI 35.0–39.9), and class III (BMI ≥ 40.0) (2). Overweight and obesity are important metabolic risk factors for various non-communicable diseases (NCDs) chronic diseases which are further responsible for approximately 75% deaths globally. In Pakistan, the overall obesity prevalence from a national level survey was 5.1% with women depicting higher burden (6.3%) as compared to men (3.2%). Furthermore, 22.8% adults were overweight, and a cumulative 27.9% prevalence was reported for both overweight and obesity (3).

Recent studies among university students in different regions of Pakistan show varying prevalence rates. A university survey reported that 23.3% of students were overweight and 6.6% were obese, while a significant proportion still remained underweight, indicating nutritional imbalance within the same population group (4). Similarly, another university based study found 10.2% of students to be overweight and 3.7% to be obese, emphasizing a consistent increase in unhealthy weight gain among students (5). Numerous investigations have recognized obesity and overweight as contributing factors for long term fatal diseases, including high blood sugar levels, hypertension and ultimately cardiovascular diseases (6). Major influencing factors in university students include, fast urban growth, physical inactivity, and increased consumption of fat and sugary products (7). Additionally, transitioning to university life and adulthood contributes to stress, often resulting in unhealthy eating patterns, such as meal skipping. Furthermore, campus life offers greater dietary autonomy, accompanied by increased intake of fast and processed foods. These poor lifestyle choices were further linked with higher obesity prevalence in university students (8). This highlights the nutrition transition happening across the globe including Pakistan, where nutritious foods are being replaced with high caloric, fat and sugar based snacks that are readily accessible to university students. Furthermore, sedentary lifestyles are highly prevalent among the younger population groups, with increased academic workload, stress, and lack of recreational facilities, all contributing to a lack of physical activity in students. A Pakistani study evidenced this association by reporting an inverse relationship between physical activity and BMI, indicating a higher body weight in those not actively engaging in any activity (9). Stress is another commonly observed contributor to increased obesity risk, as university students often face academic pressures, career uncertainties, and examination issues. Stress has been evidenced to influence appetite through physiological and endocrinal mechanisms, resulting in increased food intake among certain individuals. This is further associated with unhealthy food choices and increased BMI levels (10).

In Pakistan, the prevalence of overweight and obesity among university students is emerging as a significant public health concern. Existing literature shows an increasing trend basically impacted by unhealthy lifestyle including sedentary behavior’s, over consumption of fast or processed food and lifestyle changes like skipping major meals during university life. However, there remains a gap in current burden of obesity and overweight students in university of Islamabad and Rawalpindi. Additionally, there is a lack of clarity on whether sociodemographic variables such as age, sex, university type, household income, physical activity, and dietary behaviors, are associated with obesity in this population. Furthermore, public and private universities differ in contextual factors such as students’ socioeconomic profiles, accessibility and availability of various foods, and recreational facilities, and overall food environments, all of which may influence obesity risk. Therefore, this study aimed to estimate the prevalence of underweight, overweight, and obesity among students recruited from selected public and private universities in Islamabad and Rawalpindi and to examine sociodemographic and lifestyle factors associated with overweight and obesity.

## 2 Materials and methods

### 2.1 Study design

The study employed a cross-sectional research design to collect socio-demographic, anthropometric, and lifestyle related data from the study participants. The study was conducted and reported in accordance with the STROBE guidelines for cross-sectional studies.

### 2.2 Study area

The study was conducted in 12 public and private universities of the twin cities in Pakistan from October 10, 2025 to March 13, 2026. All 12 universities were selected via convenience sampling and to maintain institutional and individual anonymity the universities are not named within the manuscript.

### 2.3 Study population

The study population consisted of both male and female participants aged 18 years and above, studying in a public or private university, and who consented to be a part of the study. Biological sex was recorded as male or female, and there was no upper age limit to participate in the study. Pregnant and lactating females were excluded, as well as participants who were unable to provide accurate or complete information. Participants with missing data in their forms were also excluded from the study, and were enrolled only once in the study to avoid repeat participation. Medical conditions were included in the data collection questionnaire, and their potential association with obesity was considered in the analysis.

### 2.4 Sampling technique

The study employed convenience sampling for data collection, as probability sampling was not feasible due to time and resource constraints. Despite the study population being defined, access to sampling frames in both public and private universities was limited. Hence, students willing to and consenting to be a part of the study were recruited. This allowed for student participation across 12 universities, while acknowledging that this approach limited generalizability of the observed findings.

A total of 509 participants were invited to be a part of the study across the 12 selected public and private universities of Islamabad and Rawalpindi. Of the invited 509 participants, 7 declined participation, and no further exclusions were made once participants provided consent. Therefore, a total of 502 participants were a part of the study, as shown in Figure 1.

**Figure 1:**
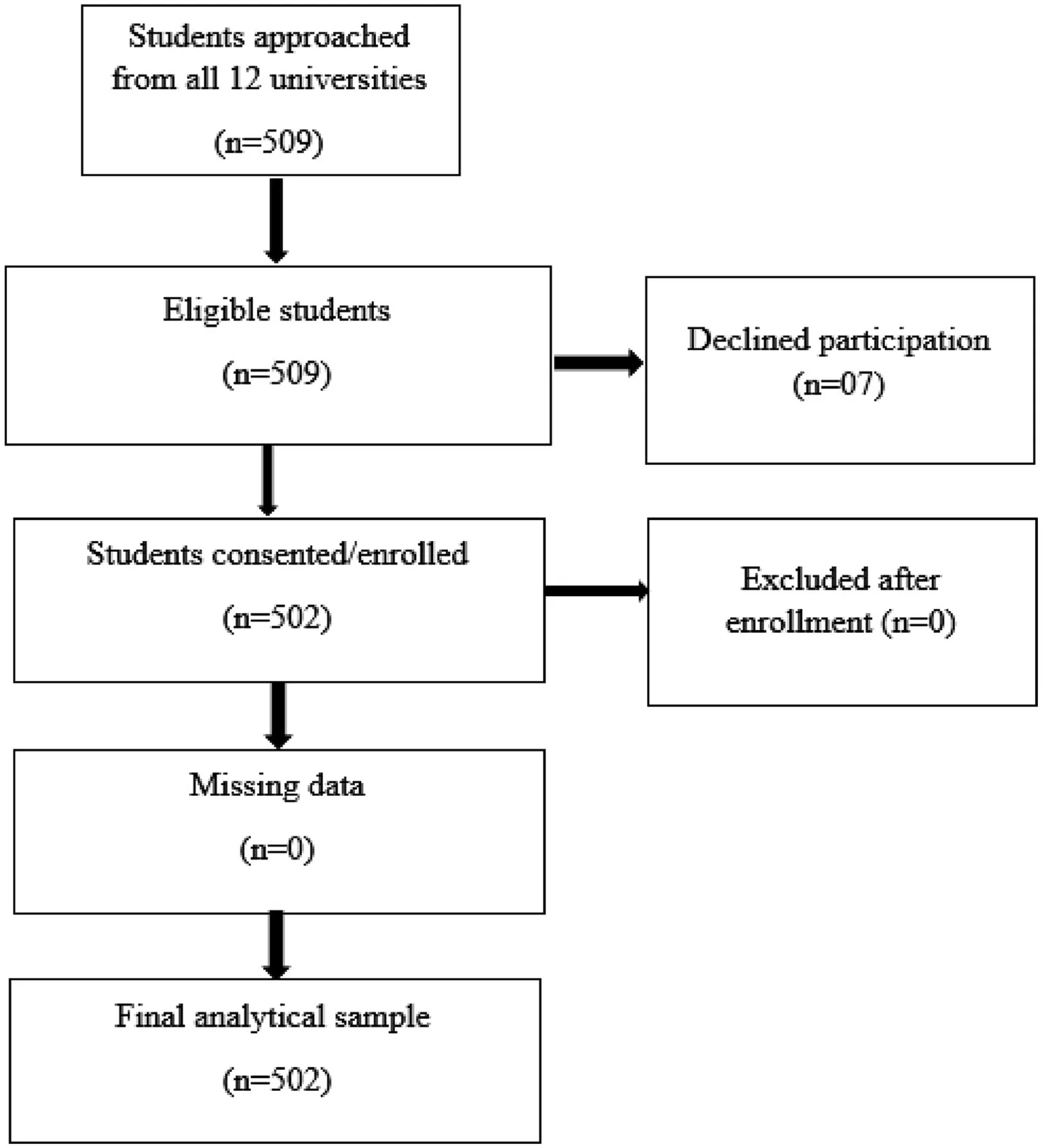
Participant flow diagram showing recruitment, participation, exclusions, and final analytical sample of university students.

### 2.5 Sample size calculation

The sample size for the study was based on a comparable cross-sectional research conducted in university students of Gujrat, Pakistan, which collected data from 432 students (5). In the current study a 15% non-response rate was employed, as commonly reported by cross-sectional researches. The adjusted sample size was calculated as 432/(1–0.15) = 508.2, which was rounded up to 509 participants, of which 502 consented to be a part of the study.

### 2.6 Data collection

Data was collected through open recruitment at the selected universities and identical data collection procedures were followed at all sites to ensure consistency of results. Data was obtained using a structured questionnaire on socio-demographic variables, anthropometric measures, and lifestyle related factors including dietary intake, physical activity levels, tobacco consumption and health status. The questionnaire was specifically developed for the current study, and the socio-demographic variables for the study included age, gender, monthly household income, paternal and maternal education, field of study, marital status, semester of study, residence status (hostel resident/day scholar), type of university (public/private), and number of siblings. These variables are among the ones that previous researches have examined among university students in association with obesity (11, 12).

The questionnaire was pilot tested on 30 university students who were not a part of the final sample. This pilot testing ensured the questionnaire’s feasibility, sequencing of questions, clarity, and comprehensibility among university students. Any identified ambiguities in questions/variables and implementation were rectified. The questionnaire was administered in both English and Urdu languages, and as the questionnaire was developed in both languages a forward and backward translation was not necessary. The questionnaire was self-administered with trained staff available on site to clarify any ambiguities.

Anthropometric measures were obtained using standardized procedures, using calibrated equipment. Nutrition graduate students were trained for anthropometric data collection according to standardized protocols. They were further trained in administration of dietary recall. The training included a practical demonstration and supervised practice to ensure measurement consistency. The trained data collectors followed the same data collection procedures across universities. These trainers directly obtained the height and weight measurements and subsequently calculated BMI, as follows:

#### 2.6.1 Weight

It was recorded using a calibrated digital weighing scale (Omron HBF-514C®, Omron Healthcare, Japan). The scale was calibrated according to the manufacturer’s instructions and was calibrated every day before starting measurements. Participants were briefed about the procedure and consent was obtained before taking their weight. They were asked to step on the center of the scale bare feet, and stand still with their body weight evenly distributed and head facing forward, while wearing minimal clothing. During data collection, the weighing scale was consistently monitored and calibrated as needed. Two readings were recorded from each participant to minimize error and weight was recorded to the nearest 0.1 kg (13). The mean of the two readings was used for analysis.

#### 2.6.2 Height

Standing height for the participants was recorded using a calibrated stadiometer (SECA 217®, SECA, Germany). Participants were asked for consent before recording their height. Once consent was obtained, they were asked to step barefoot, with minimal clothing, onto the scale with their heels together and arms hanging to the sides. Their head was positioned in the Frankfort horizontal plane, while the back of their head, shoulders, and heels were vertically aligned with the back of the stadiometer. As the participants maintained an upright posture, the head piece was lowered height was recorded to the nearest 0.1 cm. Readings were taken twice to mitigate any recording errors, and the average value was used for analysis. The stadiometer was periodically calibrated to ensure accuracy of readings (14).

#### 2.6.3 Body mass index

BMI was calculated for each participant using their measured weight and height, according to the following formula:


$$\mathrm{BMI}=\text{Weight}\;(\mathrm{kg})/\text{Height}\;{\left(m\right)}^{2}$$

Weight measured in kilograms (kg) as divided by height in meters squared (m^2^) to get BMI as kg/m^2^ and was used to assess the prevalence of overweight and obesity in university students (15). BMI was classified using the Asian specific thresholds, because European thresholds, despite being used widely in public health research globally, do not accurately portray health risks for South Asians, hence Asian specific categorization is used for these ethnic groups. The adjusted WHO BMI thresholds categorize South Asians as overweight beginning at BMI ≥ 23.0 kg/m^2^ and obese at BMI ≥ 25.0 kg/m^2^. This suggests that South Asians experience heightened NCDs risk factors at lower BMI levels as compared to the other ethnic groups (16). Therefore, to accurately determine overweight and obesity prevalence in our study Asian specific BMI cutoffs were employed.

#### 2.6.4 Dietary and lifestyle variables

Dietary variables included daily breakfast consumption, number of meals consumed per day, and junk food consumption. Daily breakfast consumption was considered if the participant consumed breakfast on all mornings of the week. Number of meals were defined as the total number of meals excluding snacks, consumed in a 24 h time period. Junk food was referred to any food that was prepared commercially and/or packaged foods that are high in energy, fats, sugars, salt, such as fast foods, packaged snacks, and sugar sweetened beverages. Daily junk food consumption was recorded as the number of times the aforementioned foods were consumed in a 24 h time period. Physical activity was self-reported regarding duration of physical activity per week and categorized as <30, 30–60, 60–120, and >120 min per week based on participants’ reported duration of physical activity. Medical condition was defined as any currently diagnosed medical condition self-reported by the participants. Tobacco consumption was defined as use of any tobacco product by the participant at the time of the survey. Similarly, alcohol consumption referred to the intake of any alcoholic beverages by the participants at the time of data collection. Both measures were self-reported by the participants.

### 2.7 Study variables

#### 2.7.1 Primary outcome variable

Body Mass Index (BMI) was the primary outcome variable for the study, to identify overweight and obese participants. It is the most widely used anthropometric measure employed to determine nutritional status and fat accumulation levels in the body. It has applications at both the individual and population level, due to its ease of calculation. It is used as a standard anthropometric measure in both observational studies and clinical trials as it offers a practical approach to determine relative adiposity levels for wider health risk assessment, while facilitating comparison across various population groups (17).

The present study did not specify any secondary outcome variables.

#### 2.7.2 Explanatory variables

These included sociodemographic variables, dietary habits, physical activity, substance use and health status of the study participants. The sociodemographic variables included age, biological sex, monthly household income, paternal and maternal education, field of study, marital status, semester of study, residence status (hostelite/day scholar), university type (public/private), and number of siblings. Age was recorded in two groups; ≤22 years and >22 years, based on prior research among South Asian university students, which also reported similar cut-offs (18).

Lifestyle and behavioral variables included breakfast consumption, number of meals per day, junk food consumption, physical activity, use of sports/exercise facilities, screen time, tobacco use, and alcohol use. Health-related variables included the presence of a medical condition and history of fractures. These factors were assessed due to the multifactorial nature of overweight and obesity and the fact that these conditions are driven by multiple modifiable risk factors. These factors may then cluster together to contribute to elevated adiposity at the population level. Hence, these variables, alongside anthropometric indicators provide a holistic approach toward obesity assessment and future intervention planning for its prevention and management (19).

#### 2.7.3 Potential confounding variables

These variables included gender, age, university type, monthly household income, physical activity, daily junk food consumption, and screen time. These variables were accounted for their potential impact on obesity, via binary logistic regression model. Age and gender were retained in the adjusted model irrespective of their statistical significance in the univariate analysis.

### 2.8 Statistical analysis

Participation was entirely voluntary; no incentives were offered and anonymity was maintained to minimize participation bias. A total of 509 students were invited to participate of whom 502 were connected (response rate = 98.6%). None of the questionnaire were excluded because of substantial missing response. All returned questionnaires were reviewed for completeness before data entry. Questionnaires containing substantial missing or inconsistent responses were excluded from the analysis as only complete questionnaires were opted for analysis, no missing data imputation were required. To minimize selection bias, students from different academic years were approached. Participation was entirely voluntary; anonymous and independent of academic evaluation thereby reducing the likelihood of coercion and encouraging honest responses. Obesity was classified on the basis of Asian BMI cut-offs (≥25 kg/m2), and were treated as separate binary outcome in the subsequent regression analysis. For each of the outcome. Obese participants were coded as 1 whereas non-obese participants were coded as 0.

Data analysis was performed by IBM SPSS Statistics v27 (IBM Corp., Armonk, NY, United States). Prior to the analysis, the data were checked for completeness, coding consistency and implausible values. Normality of quantitative variables were checked by Kolmogorov–Smirnov test. Mean± standard deviation/ median (inter-quartile range) was calculated for quantitative variables as appropriate. Frequency and percentages were reported for qualitative variables. Chi-square/fisher exact test (expected count<5) was applied to determine association between qualitative variables. Odds were calculated by binary logistic regression. Adjusted odds were calculated on the basis of clinical basis and Model fit was assessed using the Hosmer–Lemeshow goodness-of-fit test, with the final model demonstrating adequate fit (*p* = 0.295). Formal multicollinearity diagnostics based on variance inflation factors were not performed because the multivariate model primarily comprised categorical variables with no variables representing the same underlying construct or highly overlapping measurement. Variables for multivariate were selected on the basis of clinical relevance and their association with the outcome and reduant variables were not simultaneously included in the final model. The reference category was the non-obese cohort. Reference categories for each of the categorical variables are specified in their respective tables.

### 2.9 Ethical considerations

The ethical approval of the study was taken from the Institutional Review Board & Ethical Committee of the National University of Medical Sciences (NUMS), Rawalpindi, Pakistan vide letter no. 06/IRB&EC/NUMS/81. Moreover, informed written consent was obtained from all the study participants. All the research protocols were carried out in accordance with the Declaration of Helsinki. Informed consent was taken from each participant prior to their recruitment in the study.

## 3 Results

### 3.1 Socio-demographic profile of study participants

Of the 502 participants, majority (80.7%) were females, 69.1% were ages 22 years or less, 24.7% were majors in health sciences, and 73.1% were enrolled in public sector universities, as shown in Table 1. The mean age of the sample was found to be 21.87 ± 2.27. In terms of economic status, 22.1% had a family income of 50,000 to 100,000 PKR, while 31.9% students’ fathers had a bachelor’s degree, while 29.9% mothers had a bachelor’s degree.

**Table 1:** Socio-demographic profile of study participants (*n* = 502).

Variables	*n* (%)
Gender	
Female	405 (80.7)
Male	97 (19.3)
Age(years); mean ± std. dev	21.87 ± 2.27
Age group	
≤22 years	347 (69.1)
>22 years	155 (30.9)
Marital status	
Single	443 (88.2)
Engaged	33 (6.6)
Married	26 (5.2)
Degree level	
Undergraduate	452 (90)
Post graduate or above	50 (10)
Field/specialty	
Nutrition/dietetics	108 (21.5)
Health sciences	124 (24.7)
Biological and physical sciences	51 (10.2)
Engineering and IT	58 (11.6)
Social sciences and humanities	89 (17.7)
Business, law and agriculture	26 (5.2)
Other	46 (9.2)
Semester	
Semester-I	81 (16.1)
Semester-II	56 (11.2)
Semester-III	89 (17.7)
Semester-IV	68 (13.5)
Semester-V	43 (8.6)
Semester-VI	58 (11.6)
Semester-VII	38 (7.6)
Semester-VIII	59 (11.8)
Semester-IX or above	10 (2.0)
University type	
Public sector	367 (73.1)
Private sector	135 (26.9)
Hostelite/day scholar	
Hostelite	203 (40.4)
Day Scholar	299 (59.6)
Number of siblings	
None	6 (1.2)
1–3 siblings	281 (56)
>3 siblings	215 (42.8)
Monthly household income	
Less than 50 k	30 (6)
50 to 100 k	111 (22.1)
100 to 150 k	104 (20.7)
150 to 200 k	81 (16.1)
200 to 250 k	58 (11.6)
250 to 300 k	60 (12)
More than 300 k	58 (11.6)
Paternal education	
Not being able to read/write	15 (3)
Primary	6 (1.2)
Secondary	48 (9.6)
Higher secondary	98 (19.5)
Bachelor’s	160 (31.9)
Master’s/M. Phil.	148 (29.5)
Ph. D. or beyond	27 (5.4)
Maternal education	
Not being able to read/write	16 (3.2)
Primary	35 (7)
Secondary	76 (15.1)
Higher secondary	138 (27.5)
Bachelor’s	150 (29.9)
Master’s/M. Phil.	83 (16.5)
Ph. D. or beyond	4 (0.8)
Parental occupation	
Job	275 (54.8)
Business	151 (30.1)
At home	19 (3.8)
Other	57 (11.4)
Maternal occupation	
Job	82 (16.3)
Business	12 (2.4)
At home	395 (78.7)
Other	13 (2.6)

### 3.2 Dietary habits, physical activity, substance use and health status of study participants

Of the 502 students in the study, 70.3% consumed breakfast daily, 35.7% had less than 2 or less meals daily, and 46.3% did less than 30 min of physical activity per week, as shown in Table 2. Additionally, 70.3% reported that they had a sports facility available on campus, however, only 27.2% of these students used the facility. Furthermore, 5.6% consumed some form of tobacco, and 9.4% had some kind of medical condition. Figure 2 shows the BMI distribution of the study sample, with 46.2% falling in the normal BMI range, followed by 25.5% underweight students, 15.3% overweight, and 12.9% in the obese category.

**Table 2:** Dietary habits, physical activity, substance use and health status of study participants.

Variables	*n* (%)
Daily breakfast	353 (70.3)
Number of meals	
≤2 meals/day	179 (35.7)
3 meals	268 (53.4)
≥4 meals	55 (11)
Daily junk food consumption	
No consumption	81 (16.1)
1–2 times/day	402 (80.1)
≥3 times/day	19 (3.8)
Physical activity	
No physical activity	174 (34.7)
<30 min/week	151 (30.1)
30–60 min/week	100 (19.9)
60–120 min/week	71 (14.1)
>120 min/week	6 (1.2)
Sports/Exercise facility available on campus	353 (70.3)
No Sports/Exercise facility	149 (29.7)
Yes	96 (19.1)
No	257 (51.2)
Number of hours spent on screen	
Less than 1 h	9 (1.8)
1 to 2 h	42 (8.4)
2 to 3 h	98 (19.5)
3 to 4 h	126 (25.1)
More than 4 h	227 (45.2)
Current tobacco use	28 (5.6)
Current alcohol use	4 (0.8)
Medical condition	47 (9.4)
History of fractures	63 (12.5)

**Figure 2:**
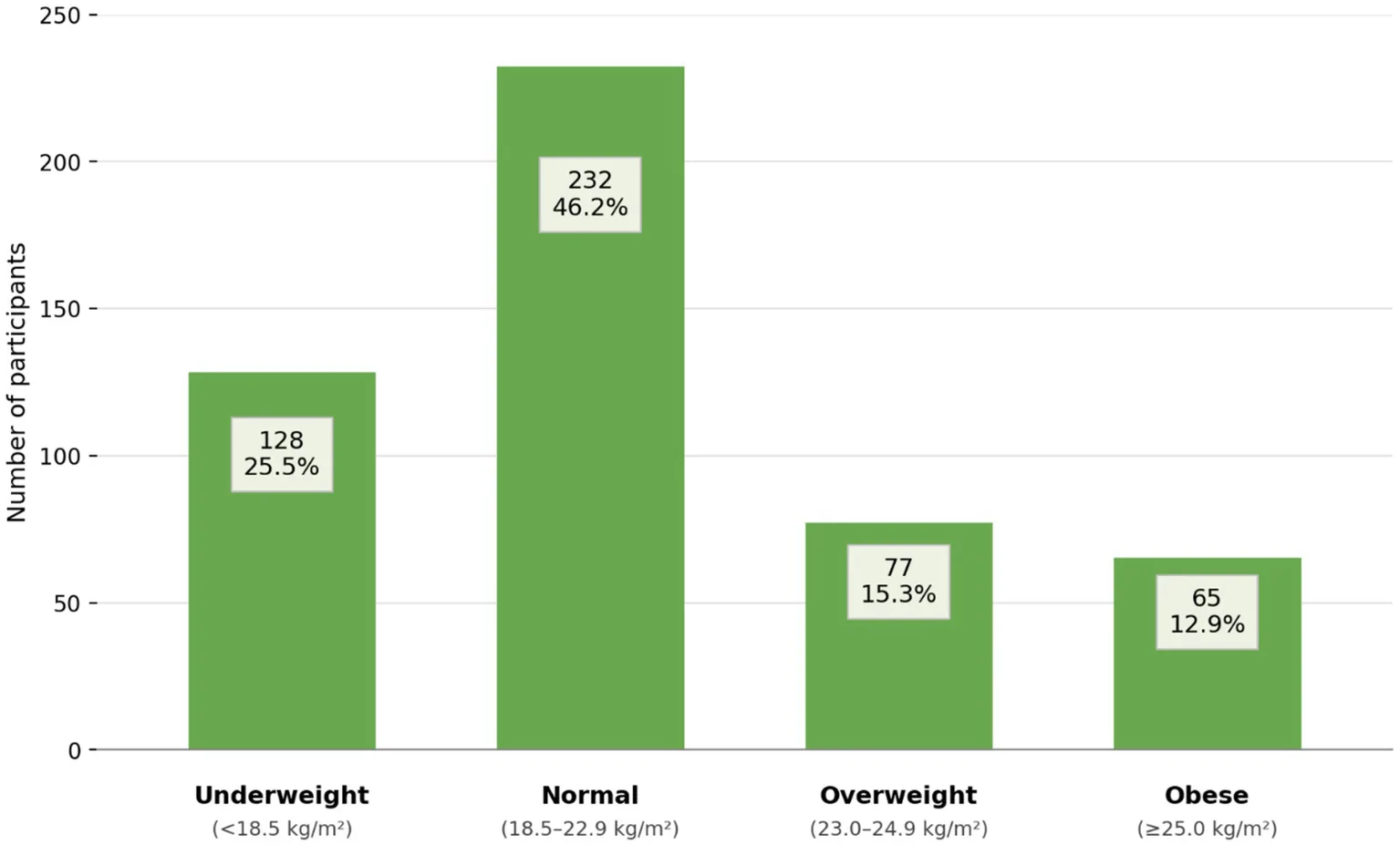
Percentage and frequency distribution of study participants on the basis of Body Mass Index (BMI).

### 3.3 Associative relationship between obesity and socio-demographic profile of study participants

Gender (*p* < 0.001), semester of study (*p* = 0.049) and household income (*p* = 0.043) were significantly associated with prevalence of obesity in university students, as shown in Table 3.

**Table 3:** Association of obesity with socio-demographic profile of study participants.

Variables	Obesity *n* (%)		*p*-value
	Obese (*n* = 65)	Non-obese (*n* = 437)	
Gender			
Female	42 (10.4)	363 (89.6)	<0.001*
Male	23 (23.7)	74 (76.3)	
Age group			
≤22 years	42 (12.1)	305 (87.9)	0.399
>22 years	23 (14.8)	132 (85.2)	
Marital status			
Single	7 (21.2)	26 (78.8)	0.168
Engaged	53 (12.0)	390 (88)	
Married	5 (19.2)	21 (80.8)	
Degree level			
Undergraduate	56 (12.4)	396 (87.6)	0.262
Post graduate or above	9 (18.0)	41 (82)	
Field/specialty			
Nutrition/dietetics	9 (8.3)	99 (91.7)	0.125
Health sciences	12 (9.7)	112 (90.3)	
Biological and physical sciences	11 (21.6)	40 (78.4)	
Engineering and IT	9 (15.5)	49 (84.5)	
Social sciences and humanities	15 (16.9)	74 (83.1)	
Business, law and agriculture	5 (19.2)	21 (80.8)	
Other	4 (8.7)	42 (91.3)	
Semester			
Semester-I	7 (8.6)	74 (91.4)	0.049*
Semester-II	12 (21.4)	44 (78.6)	
Semester-III	8 (9.0)	81 (91.0)	
Semester-IV	8 (9.0)	60 (88.2)	
Semester-V	1 (2.3)	42 (97.7)	
Semester-VI	9 (15.5)	49 (84.5)	
Semester-VII	6 (15.8)	32 (84.2)	
Semester-VIII	13 (22.0)	46 (78.0)	
Semester-IX or above	1 (10.0)	9 (90.0)	
University type			
Public Sector	44 (12.0)	323 (88.0)	0.291
Private Sector	21 (15.6)	114 (84.4)	
Hostelite/day scholar			
Hostelite	27 (13.3)	176 (86.7)	0.846
Day Scholar	38 (12.7)	261 (87.3)	
Number of siblings			
None	1 (16.7)	5 (83.3)	0.865
1–3 siblings	36 (12.8)	245 (87.2)	
>3 siblings	28 (13.0)	187 (87.0)	
Monthly household income			
Less than 50 k	4 (13.3)	26 (86.7)	0.043*
50 to 100 k	6 (5.4)	105 (94.6)	
100 to 150 k	17 (16.3)	87 (83.7)	
150 to 200 k	16 (19.8)	65 (80.2)	
200 to 250 k	5 (8.6)	53 (91.4)	
250 to 300 k	7 (11.7)	53 (88.3)	
More than 300 k	10 (17.2)	48 (82.8)	

Chi-square/fisher exact test were applied. *p*-value≤0.05 were considered as significant. ^*^Significant at 0.05 levels.

Further, binary logistic regression analysis depicted significant association between gender (*p* < 0.001) and obesity with females showing lower odds of obesity, as shown in Table 4. Participants from biological and physical sciences had the highest odds of obesity (OR = 3.025, 95% CI: 1.165–7.856, *p* = 0.023) as compared to nutrition students. Additionally, participants with a monthly household income of 50-100 k had significantly lower odds of obesity (OR = 0.274, 95% CI: 0.094–0.798, *p* = 0.018), as compared to those with an income of 300 k per month.

**Table 4:** Odds for obese patients according to socio-demographic profile of study participants.

Variables	*p*-value	OR (95% CI)
Gender		
Female	<0.001*	0.372 (0.211-0.656)
Male		1.000
Age group		
≤22 years	0.400	0.790 (0.457–1.367)
>22 years		1.000
Marital status		
Single	0.851	1.131 (0.313–4.082)
Engaged	0.280	0.571 (0.207–1.578)
Married		1.000
Degree level		
Undergraduate	0.265	0.644 (0.297–1.397)
Post graduate or above		1.000
Field/specialty		
Health sciences	0.722	1.179 (0.477–2.915)
Biological and physical sciences	0.023*	3.025 (1.165-7.856)
Engineering and IT	0.162	2.020 (0.754–5.412)
Social sciences and humanities	0.074	2.230 (0.925–5.373)
Business, law and agriculture	0.113	2.619 (0.797–8.612)
Other	0.941	1.048(0.306–3.591)
Nutrition/dietetics		1.000
Semester		
Semester-I	0.886	0.851 (0.094–7.734)
Semester-II	0.416	2.455 (0.282–21.334)
Semester-III	0.916	0.889 (0.099–7.942)
Semester-IV	0.871	1.200 (0.134–10.762)
Semester-V	0.292	0.214 (0.012–3.756)
Semester-VI	0.652	1.653 (0.186–14.694)
Semester-VII	0.647	1.687 (0.179–15.890)
Semester-VIII	0.396	2.543 (0.295–21.962)
Semester-IX or above		1.000
University type		
Public Sector	0.293	0.739 (0.422–1.297)
Private Sector		1.000
Hostelite/day scholar		
Hostelite	0.846	1.054 (0.621–1.788)
Day scholar		1.000
Number of siblings		
None	0.795	1.336 (0.150–11.857)
1–3 siblings	0.944	0.981 (0.578–1.666)
>3 siblings		1.000
Monthly household income		
Less than 50 k	0.636	0.738 (0.211–2.588)
50 to 100 k	0.018*	0.274 (0.094-0.798)
100 to 150 k	0.883	0.938 (0.398–2.210)
150 to 200 k	0.708	1.182 (0.493–2.831)
200 to 250 k	0.174	0.453 (0.144–1.419)
250 to 300 k	0.391	0.634 (0.224–1.797)
More than 300 k		1.000

OR; Odds ratio, CI; Confidence Interval. Binary logistic regression were applied. *p*-value ≤0.05 were considered as significant. *Significant at 0.05 levels.

### 3.4 Associative relationship between obesity, parental education, and occupation

Statistically significant association was found between obesity and maternal education (*p* = 0.030), while paternal education and parental occupation status remained non-significant factors associated with obesity, as shown in Table 5.

**Table 5:** Association of obesity with parental education and occupation.

Variables	Obesity		*p*-value
	Obese (*n* = 65)	Non-obese (*n* = 437)	
Paternal education			
Not being able to read/write	1 (6.7)	14 (93.3)	0.294
Primary	1 (16.7)	5 (83.3)	
Secondary	9 (18.8)	39 (81.3)	
Higher Secondary	10 (10.2)	88 (89.8)	
Bachelor’s	18 (11.3)	142 (88.8)	
Master’s/M. Phil.	19 (12.8)	129 (87.2)	
Ph. D. or beyond	7 (25.9)	20 (74.1)	
Maternal education			
Not being able to read/write	1 (6.3)	15 (93.8)	0.030*
Primary	9 (25.7)	26 (74.3)	
Secondary	5 (6.6)	71 (93.4)	
Higher secondary	16 (11.6)	122 (88.4)	
Bachelor’s	18 (12.0)	132 (88.0)	
Master’s/M. Phil.	14 (16.9)	69 (83.1)	
Ph. D. or beyond	2 (50)	2 (50)	
Parental occupation			
Job	29 (10.5)	246 (89.5)	0.219
Business	22 (14.6)	129 (85.4)	
At home	3 (15.8)	16 (84.2)	
Other	11 (19.3)	46 (80.7)	
Maternal occupation			
Job	13 (15.9)	69 (84.1)	0.553
Business	0 (0)	12 (100)	
At home	344 (87.1)	51 (12.9)	
Other	1 (7.7)	12 (92.3)	

Chi-square/fisher exact test were applied. *p*-value ≤0.05 were considered as significant. ^*^Significant at 0.05 levels.

Binary logistic regression however, showed significantly lower odds of obesity in students whose parents held a PhD or higher degree, had Higher Secondary education and a Bachelor’s degree (OR = 0.325, 95% CI: 0.110–0.957, *p* = 0.041; and OR = 0.362, 95% CI: 0.134–0.975, *p* = 0.044, respectively). Similarly, those whose mothers had a secondary or higher secondarty education had lower odds of obesity as compared to those whose mothers had a PhD or higher degree (OR = 0.070, 95% CI: 0.008–0.610, *p* = 0.016; OR = 0.131, 95% CI: 0.017–0.997, *p* = 0.050; Table 6).

**Table 6:** Univariable associations of sociodemographic factors with overweight/obesity among participating students.

Variables	*p*-value	OR (95% CI)
Parental education		
Not being able to read/write	0.158	0.204 (0.023–1.849)
Primary	0.635	0.571 (0.057–5.775)
Secondary	0.468	0.659 (0.214–2.031)
Higher Secondary	0.041*	0.325 (0.110-0.957)
Bachelor’s	0.044*	0.362 (0.134-0.975)
Master’s/M. Phil.	0.085	0.421 (0.157–1.128)
Ph. D. or beyond		1.000
Maternal education		
Not being able to read/write	0.060	0.067 (0.004–1.116)
Primary	0.322	0.346 (0.042–2.831)
Secondary	0.016*	0.070 (0.008-0.610)
Higher secondary	0.050*	0.131 (0.017-0.997)
Bachelor’s	0.053*	0.136 (0.018-1.029)
Master’s/M. Phil.	0.126	0.203 (0.026–1.564)
Ph. D. or beyond		1.000
Parental occupation		
Business	0.223	1.447 (0.799–2.619)
At home	0.481	1.591 (0.437–5.788)
Other	0.069	2.028 (0.947–4.347)
Job		1.000
Maternal occupation		
Business	0.999	NA
At home	0.478	0.787 (0.406–1.525)
Other	0.452	0.442 (0.053–3.701)
Job		1.000

OR; Odds ratio, CI; Confidence Interval. Binary logistic regression was applied. *p*-value ≤0.05 were considered as significant. ^*^Significant at 0.05 levels.

### 3.5 Associative relationship between obesity, dietary habits, physical activity, substance use and health status

None of the lifestyle factors did not report a statistically significant association with obesity, as shown in Table 7, as breakfast consumption, number of meals per day, overall junk food intake, physical activity, screen time were all found to be *p* > 0.05. Statistically significant associations were only found between having a medical condition (*p* = 0.025), and obesity.

**Table 7:** Association of obesity with dietary habits, physical activity, substance use and health status.

Variables	Obesity *n* (%)		*p*-value
	Obese (*n* = 65)	Non-obese (*n* = 437)	
Daily breakfast			
Yes	44 (12.5)	309 (87.5)	0.619
No	21 (14.1)	128 (85.9)	
Number of meals			
≤2 meals/day	22 (12.3)	157 (87.7)	0.939
3 meals	36 (13.4)	232 (86.6)	
≥4 meals	7 (12.7)	48 (87.3)	
Daily junk food consumption			
No Consumption	14 (17.3)	67 (82.7)	0.411
1–2 times/day	49 (12.2)	353 (87.8)	
≥3 times/day	2 (10.5)	17 (89.5)	
Physical activity			
No physical activity	29 (16.7)	145 (83.3)	0.121
<30 min/week	12 (7.9)	139 (92.1)	
30–60 min/week	12 (12)	88 (88)	
60–120 min/week	12 (16.9)	59 (83.1)	
>120 min/week	0 (0)	6 (100)	
Sports/exercise facility available on campus			
No Sports/Exercise facility available	19 (12.8)	130 (87.2)	0.668
Yes	10 (10.4)	86 (89.6)	
No	36 (14)	221 (86)	
Number of hours spent on screen			
Less than 1 h	0 (0)	9 (100)	0.246
1 to 2 h	4 (9.5)	38 (90.5)	
2 to 3 h	9 (9.2)	89 (90.8)	
3 to 4 h	14 (11.1)	112 (88.9)	
More than 4 h	38 (16.7)	189 (83.3)	
Current Tobacco use (In Any form)			
Yes	7 (25)	21 (75)	0.074
No	58 (12.2)	416 (87.8)	
Current alcohol use			
Yes	0 (0)	4 (100)	1.000
No	65 (13.1)	433 (86.9)	
Medical condition			
Yes	11 (23.4)	36 (76.6)	0.025*
No	54 (11.9)	401 (88.1)	
History of fractures			
Yes	13 (20.6)	50 (79.4)	0.052
No	52 (11.8)	387 (88.2)	

Chi-square/fisher exact test were applied. *p*-value ≤0.05 were considered as significant. ^*^Significant at 0.05 levels.

Binary logistic regression also showed no statistically significant association between obesity prevalence and any lifestyle factor apart from having a medical condition (*p* = 0.028), as shown in Table 8. Regression analysis found that participants with a medical condition had higher odds of being obese as compared with those reporting no diagnosed medical issue (OR = 2.269, 95% CI: 1.091–4.721, *p* = 0.028).

**Table 8:** Univariable associations of dietary and lifestyle factors with overweight/obesity among participating students.

Variables	*p*-value	OR (95% CI)
Daily breakfast		
Yes	0.620	0.868 (0.496–1.518)
No		1.000
Number of meals		
≤2 meals/day	0.931	0.961 (0.387–2.387)
3 meals	0.888	1.064 (0.447–2.533)
≥4 meals		1.000
Daily junk food consumption		
No consumption	0.475	1.776 (0.368–8.574)
1–2 times/day	0.828	1.180 (0.265–5.263)
≥3 times/day		1.000
Physical activity		
<30 min/week	0.021*	0.432 (0.212-0.880)
30–60 min/week	0.299	0.682 (0.331–1.405)
60–120 min/week	0.964	1.017 (0.486–2.127)
>120 min/week	0.999	NA
No physical activity		1.000
Sports/exercise facility available on campus		
No Sports/Exercise facility	0.722	0.897 (0.494–1.629)
Yes	0.374	0.714 (0.339–1.501)
No		1.000
Number of hours spent on screen		
Less than 1 h	0.999	NA
1 to 2 h	0.244	0.524 (0.176–1.553)
2 to 3 h	0.080	0.503 (0.233–1.085)
3 to 4 h	0.155	0.622 (0.323–1.198)
More than 4 h		1.000
Current tobacco use (in any form)		
Yes	0.057	2.391 (0.974–5.871)
No		1.000
Medical condition		
Yes	0.028*	2.269 (1.091-4.721)
No		1.000
History of fractures		
Yes	0.055	1.935 (0.985–3.802)
No		1.000

OR; Odds ratio, CI; Confidence Interval. Binary logistic regression were applied. *p*-value ≤0.05 were considered as significant. ^*^Significant at 0.05 levels.

### 3.6 Adjusted odds ratios for factors associated with obesity

As shown in Table 9, the adjusted logistic regression model found female respondents to have significantly lower obesity odds in comparison to their male counterparts (aOR = 0.428, *p* = 0.005). No other variables emerged to be significantly associated with obesity in the adjusted analysis.

**Table 9:** Adjusted odds for obese patients (*n* = 502; outcome events = 65).

Variables	*p*-value	aOR (95% CI)
Gender		
Female	0.013*	0.455 (0.245-0.846)
Male		1.000
Age group		
≤22 years	0.656	0.874 (0.484–1.580)
>22 years		1.000
University type		
Public sector	0.587	0.840 (0.447–1.580)
Private sector		1.000
Monthly household income		
Less than 50 k	0.650	0.733 (0.191–2.805)
50 to 100 k	0.039*	0.310 (0.102-0.943)
100 to 150 k	0.853	0.918 (0.368–2.287)
150 to 200 k	0.700	1.199 (0.477–3.013)
200 to 250 k	0.228	0.484 (0.148–1.577)
250 to 300 k	0.425	0.641 (0.214–1.913)
More than 300 k		1.000
Daily junk food consumption		
No consumption	0.207	2.912 (0.553–15.322)
1–2 times/day	0.507	1.690 (0.359–7.966)
≥3 times/day		1.000
Physical activity		
<30 min/week	0.105	0.527 (0.243–1.143)
30–60 min/week	0.449	0.744 (0.345–1.602)
60–120 min/week	0.936	0.965 (0.407–2.292)
>120 min/week	0.999	NA
No physical activity		1.000
Sports/exercise facility available on campus		
No sports/exercise facility	0.705	0.882 (0.462–1.686)
Yes	0.547	0.766 (0.323–1.821)
No		1.000
Number of hours spent on screen		
Less than 1 h	0.9999	NA
1 to 2 h	0.284	0.534 (0.169–1.682)
2 to 3 h	0.054	0.449 (0.199–1.014)
3 to 4 h	0.354	0.718 (0.357–1.445)
More than 4 h		1.000

aOR; Adjusted odds ratio, CI; Confidence Interval. Binary logistic regression were applied. p-value ≤0.05 were considered as significant. ^*^Significant at 0.05 levels.

## 4 Discussion

The current study aimed to find the prevalence of obesity in university students and to determine its associative factors, including socio-demographics and various lifestyle habits. The findings reflected a high burden of unhealthy lifestyles among the study population, which aligns with similar Pakistani and South Asian findings. In our study, 83.9% of the participants consumed junk food on a daily basis, only 1.2% engaged in regular physical activity for more than 120 min per week, and 45.2% had a screen time of more than 4 h, all of which are associated with increased obesity risk. A Lahore based observational study found that high fried foods (45.6%), and bakery products (74.4%) intake, was associated with obesity, which was prevalent in 34.4% of the study population (20). Another Pakistani study surveying university students found overweight and obesity prevalence to be 8 and 13%, respectively. Additionally, 41–47% of students consumed majority of their daily calories from refined carbohydrates, while only 1% consumed fruits (21). These findings are consistent with our study’s results reflecting a dual burden of unhealthy dietary choices, and sedentary lifestyles resulting in elevated overweight and obesity prevalence. This context specific generalizability in findings strengthens the need for lifestyle modifications at the university level, to prevent the emergence of chronic diseases in young adults. In terms of BMI, our study reported a 15.3 and 12.9% prevalence of overweight and obesity in the study sample. These numbers are comparable to similar Pakistani studies, such as one from Narowal, which reported 16.9% students to be overweight, while 3.5% were obese (22). Another Pakistani study collected data on obesity prevalence and its associated risk factors among university students, and reported an overweight burden of 23.6% and obesity class I burden of 6.5%. Among the risk factors, gender, sedentary lifestyle, and family history were significantly associated with obesity and overweight prevalence (11). The reported findings from our and similar previous researches strongly reflect the nutrition and lifestyle transition in the country, with particular impacts on Pakistani youth. Despite the overall burden reported in our study being lower, it still poses a public health concern, which can further result in various chronic diseases.

Our study also reported a statistically significant gender disparity in terms of gender (*p* < 0.001), as obesity was more prevalent among males respondents. This finding was again comparable to another Lahore based study which reported a statistically higher prevalence of obesity among males as compared to female students (31% vs. 16%, *p* = 0.007) (23). Similar results were found in a Karachi based study, with 49.4% male medical students being pre-obese or obese as compared to 27.5% female students (24). These findings highlight the gender disparities in obesity prevalence with male students depicting higher susceptibility to adiposity, linked with differences in dietary and physical activity patterns, sedentary lifestyles, and other lifestyle risk factors.

In the current study, monthly household income was also associated with obesity (*p* = 0.043), which is consistent with another Pakistani study from Multan, that also found that BMI increased as household income got higher, suggesting a positive association between income and obesity measures (25). Similarly, another Pakistani study found income to be associated with several obesity indicators including BMI and weight (26). These relationships, while not causal due to the cross-sectional nature of the studies, do provide insights into how higher purchasing power may increase access to energy dense foods, thereby impacting dietary patterns.

Additionally, the current study also reported higher obesity prevalence rates in studies enrolled in non-health disciplines. These findings are supported by prior evidence suggesting a link between studying health sciences, and overall improved health and nutritional literacy, and lifestyle practices. A Peru based study found higher BMI levels in business students as compared to their peers studying health sciences degrees (*p* = 0.001). Furthermore, the health sciences students reported overall lower weight, and improved diet and physical activity scores. This was linked with increased health and nutrition education in these fields, while students in business and engineering degrees were found to have comparatively higher stress levels and sedentary lifestyles, both of which negatively impact anthropometric profiles (27). Despite these findings, literature also highlights a knowledge gap in terms of not being able to convert knowledge into healthy behaviors thereby deeming knowledge alone insufficient to foster sustainable practices.

In terms of lifestyle factors and health status no lifestyle factor was significantly associated with obesity, and only having a medical condition was significantly associated with obesity, as evidenced by a *p*-value of 0.025. This finding is consistent with another study conducted on university students which reported a significant association between morbidity and obesity, as students with at least one comorbidity had a higher prevalence of obesity (28.8%) as compared to those with no medical condition (14.9%) (28). A systematic review further reported a higher prevalence of multimorbidity among obese individuals, highlighting the bidirectional relationship that exists between the obesity and chronic medical conditions. While obesity can lead to development of further comorbidities, having medical conditions can exacerbate obesity risk due to underlying metabolic and physiological pathways. Furthermore, associated treatments, drug therapies, and lifestyle changes may also contribute to obesity development in chronically ill patients (29).

Our study found daily junk food consumption was not significantly associated with obesity (OR = 0.660, *p* = 0.207). Although students who reported daily junk food consumption had lower estimated odds of obesity, the association was not statistically significant. The finding is differs from the widely supported role of highly processed fast food in various metabolic and chronic disease (30). A study on Bangladeshi university students also reported that 47.3% of the students consumed packaged snacks and 38% consumed soft drinks, despite a 87.6% awareness level regarding their health risks. This finding again highlights the translational knowledge gap in nutritional literacy and actual practices (31). Another Lahore based university study surveying obese students found a 56% preference rate for junk food, 63% daily consumption prevalence, and 57% daily intake of sugary foods. These findings highlight the previously evidenced pattern of high junk food intake to increase or contribute toward obesity risk (32). This is scientifically plausible due to the high concentration of saturated fats, sugars, and refined carbohydrates in these foods, all of which lead to excessive caloric intake and resulting weight gain. Additionally, tobacco consumption was also not reported as a significantly associated factor with obesity in our study, which is consistent with literature stating that tobacco consumption is associated with weight loss as nicotine causes appetite loss (33). However, recent evidence has suggested a strong link between tobacco intake and adverse health impacts including obesity. A Mendelian randomization study involving 378 genetic variants found that genetically predicted smoking initiation is significantly associated with a 0.28 increase in BMI, 0.88 increase in waist circumference, and a 0.006 point increase in waist to hip ratio (34). There has been an emerging trend of e-cigarettes and vapes in university students, as evidenced by a 29.8% prevalence of some kind of smoking. Out of these 16.7% used vapes and 35.0% used traditional cigarettes. Smoking was also associated with further unhealthy practices such as poor dietary intake, sedentary lifestyles, and stress, all of which contribute to obesity (35).

However, adjusted odds only found gender to be significantly associated with obesity (*p* = 0.005) in the study sample. This highlights that the crude significance of variables such as income, and medical conditions, may have been influenced by confounding factors, such as age, physical activity, and dietary patterns. Therefore, while these variables may not be independently significantly associated with obesity, they likely cluster with other behavioral factors to increase obesity risk and prevalence in university students. Such differences in crude and adjusted odds ratio are commonly reported in observational studies, as confounders commonly influence each other.

A key strength of the current study was the use of standardized anthropometric measurements by trained data collectors, rather than reliance on self-reported data. Additionally, students from diverse academic backgrounds and both public and private sectors were included providing a wider representation of Pakistani university students, within Rawalpindi and Islamabad. Furthermore, the study assessed several lifestyle and health related factors such as dietary habits, physical activity, screen time, tobacco and alcohol use, and current medical conditions. This allowed for a comprehensive and holistic assessment of the factors associated with obesity.

The study also has some limitations that need to be acknowledged. The use of convenience sampling to recruit only participants willing and accessible, limited the representativeness of the sample and generalizability of the findings, which may not be applicable to university students across Pakistan. Future research on the topic may benefit from employing a sampling technique that improves representativeness and generalizability its findings. Furthermore, the study sample was female dominant which may have resulted in gender bias in the findings, thereby limiting their generalizability. Although, gender was adjusted for in the multivariable analysis, the findings should be interpreted with caution. Additionally, behavioral variables were self-reported which may have been subjected to recall and social desirability bias. Some measures were not based on validated instruments which may have impacted the measurement accuracy. Moreover, the cross-sectional study design may limit the establishment of causal relationships between the studies variables and obesity. Therefore, these findings should be incorporated with caution as they may not be generalizable to all university students of Pakistan.

The current study’s findings have important implications and recommendations for future research and the university environment. Universities should work on strengthening health promotion initiatives focused on lifestyle factors including healthier diets, physical activity, and reduction of unhealthy food access and availability on campus. Healthier and affordable food options in campus cafeterias may reduce dependence on unhealthy processed foods among students. Availability of recreational facilities in university including gyms and sports centers are needed to foster active lifestyles among the youth. The double burden of under and overnutrition requires a comprehensive and holistic approach to address both issues rather than solely focusing on obesity prevention. Periodic nutrition screening including anthropometric assessment, coupled with awareness sessions in universities are recommended to identify at risk populations and to develop and implement subsequent prevention and management interventions. These strategies may help mitigate the growing burden of malnutrition among university students in Pakistan.

## 5 Conclusion

The present study highlights the growing burden of overweight and obesity, as significant health concerns among university students, as evidenced by a 15.3 and 12.9% prevalence of overweight and obesity, respectively. Despite a high number of students reporting normal BMIs, the co-existence of under and overweight students adds to the emerging evidence of the double burden of malnutrition within the same population. An interplay of behavioral, environmental, and physiological factors play a role in the emergence of obesity. In the final adjusted analysis, male gender was the only factor significantly associated with obesity, however this finding should not be interpreted as a causal relationship. Our study’s results highlight the need for public health interventions at the primary level aimed at fostering healthier lifestyles among students, as well as modifying the campus environment in a way that is conducive to health. Nutrition and health awareness initiatives, facilities for recreational physical activity, and health promotion policies for students, can help mitigate the emerging trend of obesity and foster sustainable health outcomes. Given the high prevalence of underweight along with overweight and obesity highlights the double burden of malnutrition among university students. Further representative studies are needed to generalize these findings to Pakistani university students.

## Data Availability

The raw data supporting the conclusions of this article will be made available by the authors, without undue reservation.
